# Policy: Cloud Banks: Airlines Save Halon

**DOI:** 10.1289/ehp.114-a95a

**Published:** 2006-02

**Authors:** Adrian Burton

The airlines of the developing world are being advised by the United Nations Environment Programme (UNEP) to bank their stocks of halons—chemicals vital for extinguishing aircraft fires—as the 2010 deadline to cease production approaches. Most developed nations already have plans for halon recycling and banking systems—registries of who has excess halon to sell. For developing countries, however, the challenges of starting up such systems may leave some airlines grounded.

Halons have been used for years in many kinds of fire-extinguishing systems. However, when they escape into the atmosphere, UV light causes them to release highly reactive bromine radicals that deplete the ozone layer. Indeed, halons are thought to be three to ten times more ozone-unfriendly than chlorofluorocarbons. For this reason the Montréal Protocol obliged developed nations to cease halon production in 1994, and set a 2010 target date for the developing world.

The trouble is that, while replacements for halons now exist for nearly all other applications, these chemicals remain essential for aircraft safety. Jim Curlin, information manager of the UNEP Division of Technology Industry and Economics, OzonAction Branch, explains, “Aircraft fire-extinguishing systems must have good dispersion and fire-suppression functions, must work at low temperatures, be of low toxicity to humans for the time that they are trapped in an affected plane and have an excellent weight-to-volume ratio.” Currently, he says, there is no drop-in replacement for halons that has all these characteristics, making halon availability critical to airlines.

Even developed countries are not without halon banking problems. Developed nations have enough halon 1301—which is used in cargo bay and engine fire-fighting equipment—to last some 25 years, by which time a replacement should be available, explains John O’Sullivan, a member of the UNEP Halons Technical Options Committee and fire representative for the International Air Transport Association in Montréal. But there isn’t enough halon 1211, which is used by aircrew in handheld extinguishers. “[Halon 1211] can still be made in developing countries, so at least in this respect [developing nations] should have fewer problems,” says O’Sullivan. “But European regulations, for example, make it difficult to import. This is a problem we still have to address.”

Starting up halon banking systems is certainly in the best interest of developing world airlines. With passenger safety a top priority, aircraft that do not maintain their halon-based systems would eventually fail airworthiness inspections and be banned from flying to many destinations. But how easy will it be for developing countries to start such systems, and where does halon banking figure on their priority list?

“Most focus first on economic problems and then on the environment,” says Wilman Rajiman, the Indonesia Halon Bank Project manager at Soekarno-Hatta International Airport in Jakarta. “In Indonesia we started to discuss a national halon bank in 1995, but due to an economic crisis in 1998 it was not launched until March 2000. The major problems we faced were capital investment, knowledge, training, and local regulations.”

For many countries, cash flow will be the major obstacle. Rajiman explains that Indonesia received a grant from the World Bank, but must spend its own money and then ask for reimbursement. Poorer nations may find that stipulation difficult, yet airline-servicing companies worldwide must comply strictly with the halon specifications laid down by aircraft manufacturers and foreign aviation authorities. Maintaining proper halon stocks is therefore vital to their business.

Flyers may be comforted to know that the Montréal Protocol contains a clause that allows developing nations to temporarily restart halon production for critical systems if supplies fail—always supposing the necessary infrastructure exists. “That’s a situation we all want to avoid,” says Curlin, “and one of the reasons we are encouraging companies and countries to develop halon banks.”

## Figures and Tables

**Figure:**
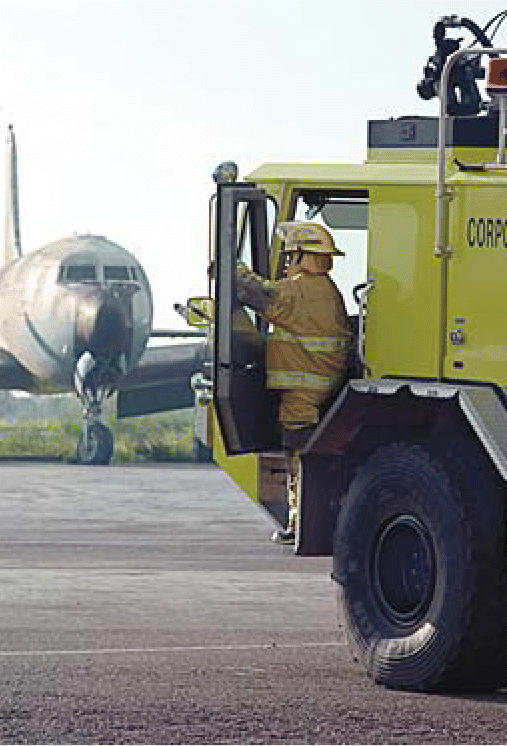
Fear of firing. Halon, used to put out fires on aircraft, is being phased out with no suitable replacement in sight.

